# P-1920. The influence of comorbidity, age, sex, and immunosuppression on COVID-19 mortality throughout the course of the SARS-CoV-2 pandemic in Europe: Data from the LEOSS study

**DOI:** 10.1093/ofid/ofae631.2080

**Published:** 2025-01-29

**Authors:** Julian Triebelhorn, Maria M Ruethrich, Susana M Nunes de Miranda, Jochen Schneider, Timm Westhoff, Margarete Scherer, Christoph Spinner, Maria J GT Vehreschild, Floran Voit, Julia Lanznaster, Johanna Erber, Kerstin Hellwig, Björn-Erik O Jensen, Laura Wagner

**Affiliations:** Department of Internal Medicine II, University Hospital rechts der Isar, Technical University of Munich, School of Medicine, Munich, Germany, Munich, Bayern, Germany; Department of Interdisciplinary Intensive Care, Vivantes Humboldt Hospital, Berlin, Berlin, Germany; University of Cologne, Faculty of Medicine and University Hospital Cologne, Department I of Internal Medicine, Center for Integrated Oncology Aachen Bonn Cologne Duesseldorf, Cologne, Nordrhein-Westfalen, Germany; TUM School of Medicine and Health, Department of Clinical Medicine – Clinical Department for Internal Medicine II, University Medical Center, Technical University of Munich, Munich, Bayern, Germany; Department of Internal Medicine I, Marien Hospital Herne Ruhr University Bochum, Herne, Nordrhein-Westfalen, Germany; Goethe University Frankfurt, Faculty of Medicine, Institute for Digital Medicine and Clinical Data Science, Frankfurt, Hessen, Germany; Department of Medicine II, University Hospital rechts der Isar, Technical University of Munich, Munich, Germany, Munich, Bayern, Germany; Department of Internal Medicine, Infectious Diseases, University Hospital Frankfurt Goethe University Frankfurt, Frankfurt am Main, Hessen, Germany; Department of Internal Medicine II, University Hospital rechts der Isar, Technical University of Munich, School of Medicine, Munich, Germany, Munich, Bayern, Germany; Department of Internal Medicine II, Hospital Passau, Passau, Bayern, Germany; Technical University of Munich, School of Medicine – University Hospital, Department of Internal Medicine, Gastroenterology, Munich, Bayern, Germany; Department of Neurology, St. Josef-Hospital Bochum, Ruhr University Bochum, Bochum, Nordrhein-Westfalen, Germany; Department of Gastroenterology, Hepatology and Infectiology, Heinrich-Heine-University, Duesseldorf, Germany, Düsseldorf, Nordrhein-Westfalen, Germany; TUM School of Medicine and Health, Department of Clinical Medicine – Clinical Department for Internal Medicine II, University Medical Center, Technical University of Munich, Munich, Bayern, Germany

## Abstract

**Background:**

Population immunity against SARS-CoV-2 has increased, resulting in a decline of COVID-19 mortality. The aim of the study was to analyse the COVID-19-related mortality over the pandemic, stratified by a model of groups at risk of severe COVID-19.

Predicted probability of COVID-19-related death in dependence of age and SARS-CoV-2 variant.

**Figure:**
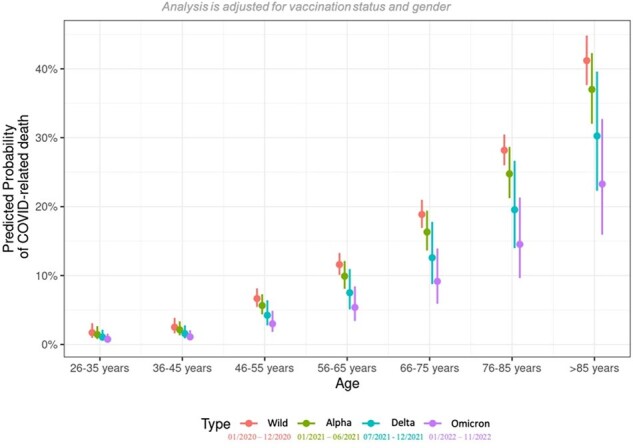

**Methods:**

COVID-19-related patients were included from January 2020 to November 2022 using the international multicentric cohort study Lean European Open Survey on SARS-CoV-2-Infected Patients (LEOSS). Predicted probability of COVID-19-related death was calculated using a multivariate logistic regression model adjusted to age, gender and vaccination status.

**Figure 2:**
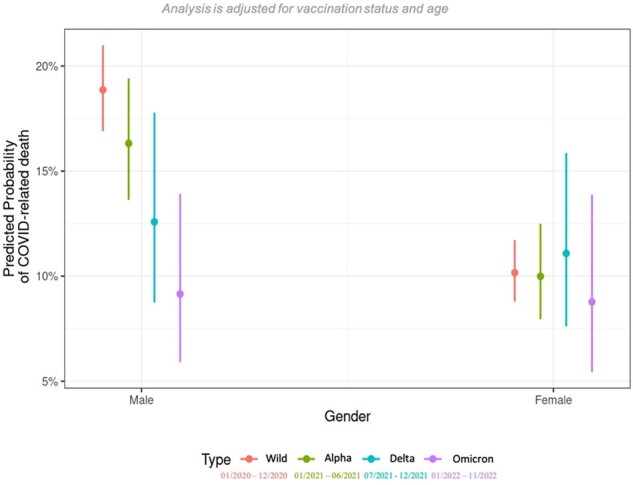
Predicted probability of COVID-19-related death in dependence of gender and SARS-CoV-2 variant.

**Results:**

In total, 12,096 patients were included. The overall mortality was 13% (n=1546), decreasing from 14% during the wildtype (wt) period (01/2020-12/2020), to 13%, 10%, and 6% in the alpha (α) (01/2021-06/2021), delta (δ) (07/2021-12/2021), and omicron (Ω) (01/2022-11/2022) periods, respectively. Patients aged 66-75, 76-85, and >85 years (y) had a 13.3-, 22.5-, and 40.4-fold higher odds of mortality compared to 26-35y old patients (p< 0.001 in all listed comparisons). This increase in mortality between younger (age: 26-35y, mortality: wt 2%, Ω 1%) and older patients (age: >85y, mortality: wt 41%, Ω 23%) decreased with the shift from wt (increase of 39 percentage points) to Ω (increase of 22 percentage points) (Fig. 1). The overall mortality in males (m) (15%) was higher than in females (f) (10%), but this gender-specific difference leveled off with the shift from wt (m: 19%, f: 10%) to Ω (m: 9%, f: 9%) (Fig. 2). Referring to the difference in mortality between patients with zero and four comorbidities, the predicted increase in mortality during the periods wt, α, δ, and Ω was 14, 14, 1, and 0 percentage points, respectively (Fig. 3). Concerning severely immunosuppressed patients, mortality significantly decreased throughout the pandemic (wt: 15%, Ω: 4%, p=0.031) (Fig. 4).

**Figure 3:**
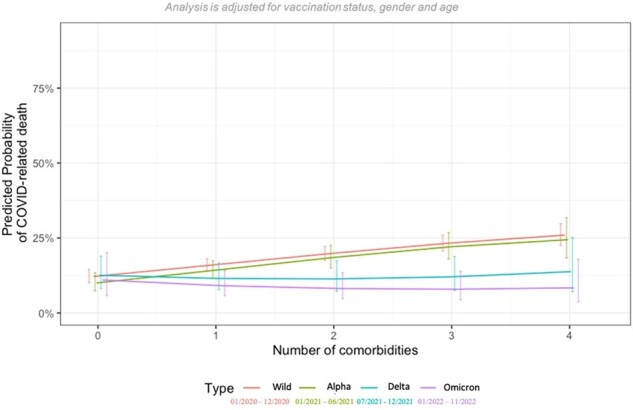
Predicted probability of COVID-19-related death in dependence of number of comorbidities and SARS-CoV-2 variant. Comorbidities include cardiovascular diseases, renal diseases, diabetes, pulmonary diseases, liver diseases, rheumatic diseases, haemato-oncological diseases, neurological diseases, autoimmune diseases and HIV/AIDS.

**Conclusion:**

Overall mortality decreased during the pandemic. This change was observed even among severely immunosuppressed patients. Age, gender, and the number of comorbidities were identified as relevant mortality risk factors, with decreasing importance as the pandemic progressed.

**Figure 4:**
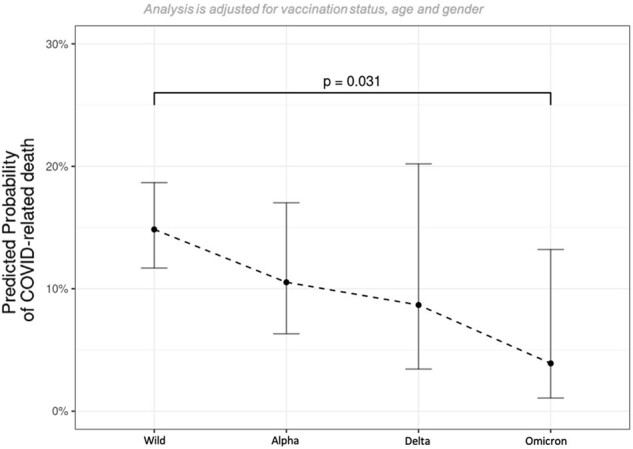
Subgroup analysis for severely immunosuppressed patients. Severely immunosuppressed group, including: stem cell transplant, organ transplant, b-cell depletion, autoimmune disease, immunosuppressive therapy (corticosteroids, methotrexate, azathioprine, ciclosporin, calcineurin-inhibitors, mTOR-inhibitors).

**Disclosures:**

Jochen Schneider, n/a, AbbVie: Grant/Research Support|AbbVie: Honoraria|AbbVie: non-financial support|Central Innovation Program for small and medium-sized enterprises: Grant/Research Support|Central Innovation Program for small and medium-sized enterprises: Honoraria|Central Innovation Program for small and medium-sized enterprises: Central Innovation Program for small and medium-sized enterprises|Deutsche Forschungsgemeinschaft: Grant/Research Support|Deutsche Forschungsgemeinschaft: Honoraria|Deutsche Forschungsgemeinschaft: non-financial support|Diasorin: Grant/Research Support|Diasorin: Honoraria|Diasorin: non-financial support|Dr. Falk Pharma GmbH: Grant/Research Support|Dr. Falk Pharma GmbH: Honoraria|Dr. Falk Pharma GmbH: non-financial support|Gilead Sciences: Grant/Research Support|Gilead Sciences: Honoraria|Gilead Sciences: non-financial support|GSK/ViiV Healthcare: Grant/Research Support|GSK/ViiV Healthcare: Honoraria|GSK/ViiV Healthcare: non-financial support|Janssen-Cilag: Grant/Research Support|Janssen-Cilag: Honoraria|Janssen-Cilag: non-financial support|MSD: Grant/Research Support|MSD: Honoraria|MSD: non-financial support|Takeda Pharmaceutical: Grant/Research Support|Takeda Pharmaceutical: Honoraria|Takeda Pharmaceutical: non-financial support Christoph Spinner, MD, AbbVie: Honoraria|Apeiron: Honoraria|AstraZeneca: Grant/Research Support|AstraZeneca: Honoraria|BBraun Melsungen: Honoraria|BBraun Melsungen: non-financial support|BioNTech: Honoraria|Cepheid: Grant/Research Support|Eli Lilly: Honoraria|Formycon: Honoraria|Gilead Sciences: Grant/Research Support|Gilead Sciences: Honoraria|Gilead Sciences: non-financial support|GSK: Honoraria|Janssen-Cilag: Grant/Research Support|Janssen-Cilag: Honoraria|Molecular partners: Honoraria|MSD: Grant/Research Support|MSD: Honoraria|Pfizer: Honoraria|Roche: Honoraria|Shionogi: Honoraria|SOBI: Honoraria|Synairgen: Honoraria|ViiV Healthcare: Grant/Research Support|ViiV Healthcare: Honoraria Maria JGT Vehreschild, Prof. Dr. med., 3M: Honoraria|Ärztekammer Niedersachsen: Honoraria|ADKA: Honoraria|Akademie für Infektionsmedizin: Honoraria|Astellas: Honoraria|Berliner Dialy Seminar: Honoraria|Biontech: Grant/Research Support|CED Service: Honoraria|DiaLog Service: Honoraria|EUMEDICA: Advisor/Consultant|EUMEDICA: Honoraria|Falk Foundation: Honoraria|Ferring: Honoraria|Forum für medizinische Fortbildung Gmbh: Honoraria|GILEAD: Honoraria|Heel: Grant/Research Support|Helios Kliniken: Honoraria|Institute Merieux: Honoraria|Janssen: Honoraria|Kit Kongress: Honoraria|Klinikum Essen: Honoraria|Klinikum Leverkusen: Honoraria|Lahn-Dill-Kliniken: Honoraria|Landesärztekammer Hessen: Honoraria|Limbach Gruppe SE: Honoraria|MaaT: Advisor/Consultant|MSD: Advisor/Consultant|MSD: Grant/Research Support|MSD: Honoraria|Pfizer: Honoraria|Roche: Advisor/Consultant|Roche: Grant/Research Support|SD Biosensor: Grant/Research Support|St. Johannes Hospital: Honoraria|St. Josef Hospital: Honoraria|SUMIT OXFORD Ltd.: Honoraria|Tillotts: Advisor/Consultant|Tillotts: Honoraria|Uniklinik Frankfurt: Honoraria|Uniklinik Köln: Honoraria|Uniklinik Karlsruhe: Honoraria|Universitätsklinikum Freiburg: Honoraria|Universitätsklinikum Heidelberg: Honoraria Floran Voit, MD, Gilead Sciences: Honoraria|Gilead Sciences: non-financial support|MSD: Grant/Research Support|Pfizer: non-financial support|ViiV Healthcare: non-financial support Johanna Erber, Dr. med., Gilead Sciences: non-financial support|Pfizer: non-financial support|Tillotts Pharma AG: non-financial support|ViiV Healthcare: non-financial support

